# Dystrophic mineralization of the arterial fibrovascular tissue associated with a vitamin D hypervitaminosis in an 8-year-old female Alpaca (*Vicugna pacos*)

**DOI:** 10.1186/s13620-016-0078-1

**Published:** 2016-12-15

**Authors:** Alexander Tavella, Annalisa Stefani, Claudia Zanardello, Astrid Bettini, Matthias Gauly, Patrik Zanolari

**Affiliations:** 1 Istituto Zooprofilattico Sperimentale delle Venezie, viale dell’Università 10, 35020 Legnaro, Italy; 2 Libera Università di Bolzano, Department of Animal Science, Faculty of Science and Technology, Piazza Università 5, 39100 Bolzano, Italy; 3 Clinic for Ruminants, Vetsuisse-Faculty of the University of Bern, Bremgartenstrasse 109A, 3012 Bern, Switzerland

**Keywords:** Alpaca, Hypervitaminosis D, Dystrophic mineralization

## Abstract

**Background:**

Prophylactic Vitamin D supplementation is a common practice in Alpaca breeding in many regions around the world. An overdosage can lead to dystrophic mineralization of soft tissues. In this paper we illustrate a suspected case of hypervitaminosis D in an 8-year-old female Alpaca.

**Case presentation:**

In June 2015, the carcass of an 8-year-old female Alpaca (*Vicugna pacos*) was submitted to the diagnostic laboratory of the *Istituto Zooprofilattico Sperimentale delle Venezie* (IZSVe) for necropsy. The animal had undergone a spontaneous abortion with uterine prolapse and delivery of the placenta, and had died shortly thereafter. Death occurred due to internal haemorrhage related to dystrophic mineralization of the left renal artery with subsequent rupture and damage of the renal hilum. During the necropsy, histopathological and serum biochemical analyses were performed. After laboratory analyses and the history of mineral and vitamin supplementation reported by the breeder, a hypervitaminosis D was suspected to be the most probable cause of the dystrophic mineralization observed in the left renal artery.

**Conclusions:**

Most of the information regarding Llamas and Alpacas comes from the South American and Australian regions. It is therefore important to provide scientific information about these animals in other regions of the world in order to have a better and wider understanding of the nutritional and environmental conditions necessary for optimal breeding.

## Background

Hypervitaminosis D in South American camelids (SAC) is often caused by an overzealous supplementation of cholecalciferol (Vitamin D_3_). The prophylactic administration of Vitamin D_3_ is a common practice in preventing the appearance of rickets in SAC crias raised in regions characterized by prolonged periods of reduced sunlight [1, 2]. Vitamin D is generally stored in the liver; its overdose can lead to demineralization of the skeleton, hypocalcemia, hyperphosphatemia and calcinosis. Prophylactic therapy with Vitamin D_3_ is less common in adult animals than in young ones: this report describes a case of dystrophic mineralization of the renal arteries in an 8-year-old female Alpaca possibly caused by hypervitaminosis D.

## Case presentation

### History

In June 2015, a breeder who had attended a conference in New Zealand on SAC breeding, where hypovitaminosis D is of great relevance, and is well informed on SAC nutritional needs and behaviors, reported a case history of a late term abortion in an 8-year-old brown female Alpaca (8^th^ month of gestation). The strong uterine contractions caused a uterine prolapse (two years before the same animal also had a uterine prolapse after physiologic delivery). The placenta was delivered immediately after abortion, and the uterus was soon repositioned by a veterinarian and retained with a suture. The animal was observed closely after uterine reposition; paleness of the mucous membranes, apathy, lethargy and hypothermia were observed within two days. At first sight, the clinician suspected that the rupture of the large uterine arteries had caused the described clinical signs. The hembra died two days after the abortion and repositioning of the uterus. The carcass was submitted within an hour to the diagnostic laboratory of the *Istituto Zooprofilattico Sperimentale delle Venezie* (IZSVe) for necropsy.

### Necropsy findings

**Fig. 1:**
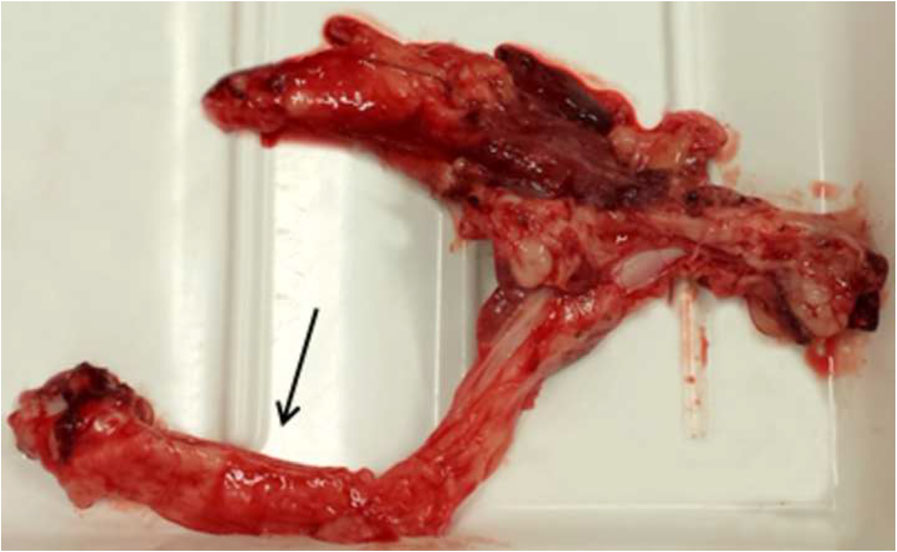
The ossified left renal artery

The alpaca had been recently sheared, and at the external examination it showed good nutritional status, weighing 61 kg; it presented with pale mucous membranes, haematoma and oedema of the external genital tract as well as a shoelace patterned suture on the vulval lips, gained during the retention of the prolapsed and repositioned uterus. No other external macroscopic lesions were recognizable. Among the gross necropsy findings, we observed haemoperitoneum, extended coagulum in the pelvic space, a sharp laceration in the region of the renal hilum of the left kidney, severe sclerosis of the renal arteries, and a diffuse dystrophic mineralization of all blood vessels in the pelvic space and in the abdomen (Fig. 1). The hardened left renal artery was broken in proximity to the renal hilum: the sharp end of the mineralized vessel had injured the hilum of the left kidney causing the intra-abdominal haemorrhage, and therefore the death of the animal.

The uterus was oedematous and haemorrhagic because of the abortion and the associated prolapse. Furthermore, a dilated chronic cardiomyopathy of the right ventricular chamber was diagnosed. Parasitic enteritis, caused by gastrointestinal strongyles, was confirmed by coprological examination (flotation and sedimentation – in house qualitative methods). No other parasites were observed. The other organs were unremarkable and no pathological alteration could be detected.

### Histopathological findings

**Fig. 2:**
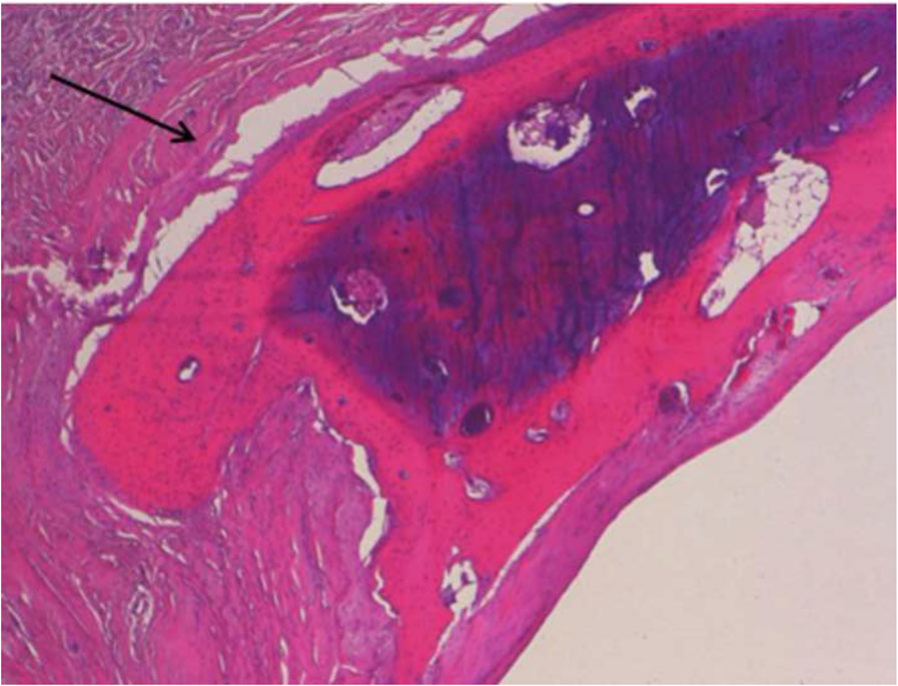
Focal ossification with dystrophic mineralization in the fibrovascular tissue of the left renal artery (HE)

Samples of both renal arteries were collected and fixed in 10% neutral buffered formalin for histological examination. The samples were processed, paraffin embedded, stained with haematoxylin and eosin (HE) and observed by standard light microscopy for histological examination. A focal dystrophic ossification was observed in the fibrovascular tissues of the left renal artery (Fig. 2).

### Serum biochemical profile

A blood sample was collected from the heart immediately after submission for necropsy (within an hour of death), centrifuged for 10 min at 3000 × g, and stored at -20 °C until analysis. The serum biochemical profile revealed hyperphosphatemia (7.07 mmol/l; range 1.1–2.8 mmol/l), hypermagnesemia (2.9 mmol/l; range 0.8–1.1 mmol/l), mild hypocalcemia (1.98 mmol/l; range 2.1–2.5 mmol/l), hyponatremia (138 mmol/l; range 148–155 mmol/l), hypochloremia (99 mmol/l; range 111–146 mmol/l), hyperuremia (17.3 mmol/l; range 4.5–9.1 mmol/l), hypercreatininemia (288 μmol/l; range 104–168 μmol/l), and a hypoproteinemia (48 g/l; range 56.2–70.4 g/l) with a mild hypoalbuminemia (27 g/l; range 28.4–37.4 g/l) and normoglobulinemia (22 g/l; range 21–31 g/l). All biochemical parameters were analyzed by the means of commercial kits (Roche Diagnostics, Mannheim, Germany) applied to the analyzer Cobas C501 (Roche Diagnostics): principles of the methods and analytical performance are described in Table 1.

**Table 1:** Serum biochemical profile

Biochemical parameter	Analytical method	Linearity	Intra-assay variation (CV%)	Inter-assay variation (CV%)
Total protein	Biuret	2.0–120 g/l	1.1	1.5
Albumin	Albumin bromocresol green (BCG)	2–60 g/l	0.7	1.0
Globulin	Calculated parameter	–	–	–
Urea	UV kinetic – Urease	0.5–40 mmol/l	2.1	3.4
Creatinine	Enzymatic – PAP	5–2700 μmol/l	1.1	1.2
Calcium	o-cresolphtalein complexone	0.20–5 mmol/l	1.0	1.6
Phosphate	Phosphomolybdate	0.10–6.46 mmol/l	0.7	1.4
Magnesium	Xylidyl blue	0.10–2.0 mmol/l	1.1	1.3
Sodium	Indirect potentiometry	80–180 mmol/l	0.3	0.5
Chlorine	Indirect potentiometry	60–140 mmol/l	0.3	0.6

Range values from live animals are in accordance with Hengrave Burri et al., [3], Dawson et al., [4], and Husakova et al., [5]. Unfortunately, no reference range values regarding dead animals are available. Furthermore, it is known that post-mortem values are frequently not reliable and difficult to interpret, because of the physiological events that happen in an organism after its death. In a work reported in 2008, Ferner describes how post-mortem changes render the assumption of clinical pharmacology largely invalid, making the interpretation of concentrations measured in post-mortem samples difficult and even impossible [6].

## Conclusions

Vitamin D_3_ is synthesized in the skin of animals by ultraviolet (UV) irradiation of 7-dehydrocholesterol; an insufficient exposure to sunlight may therefore result in a Vitamin D_3_ deficiency. According to Smith and Van Saun [7], Vitamin D_3_ concentrations in SAC vary significantly depending on the season: the lowest concentrations are measured in the months of September and October and then again in February and March, corresponding to the months before and following the period of less exposure to sunlight in central Europe [2, 7, 8, 9]. The herd is indeed located in a valley of the Dolomites (Italy) characterized by very few hours of sunlight a day (approximately 2 h of sunlight in January, 4 h in February, and 5 h in March, [10]). During the winter months, blood Vitamin D concentrations drop to approximately one third to one sixth of summer concentrations [11]. Furthermore, more darkly colored and highly fleeced animals present lower Vitamin D concentrations, although shearing increases skin exposure and therefore Vitamin D concentrations [12]. The hypothesis that SAC have adapted to the constant UV radiation in their native environment by reducing their capability of producing this vitamin [7] would most likely confirm why supplying animals with Vitamin D has positive effects on their general wellbeing, preventing possible vitamin and mineral deficiencies. Hypovitaminosis D has been suggested as the main cause of rickets in young growing SAC, and supplementation of Vitamin D seems to be an effective tool in preventing hypophosphataemic rickets syndrome [13, 14]. Prevention campaigns usually consider oral administrations of 15,000-30,000 IU of vitamin D every 2 weeks during the winter, intramuscular administration of 454 IU/kg every 3 months, or subcutaneous injections of 1000 IU/kg body weight in late autumn and mid-winter at an interval of 90 days [13, 8, 15]. There is no scientific evidence that giving higher than the above-mentioned doses of Vitamin D to lactating mothers would lead to higher concentrations of this vitamin in the milk [8], and, therefore, prophylactic therapy with Vitamin D_3_ is generally less common in adult animals.

In the described case, the hembra was treated twice with Vitamin D (Duphral® D_3_ 1000, Zoetis Italia Srl, via Andrea Doria 41 M, 00192 Roma, Italy), respectively in November 2014 and January 2015. Both times, 0.5 ml of the product were administered intramuscularly (corresponding to 500,000 IU of Vitamin D_3_ per treatment). The prescription mentioned is usually used to prevent hypocalcemia in dairy cows, according to the manufacturer’s explicit recommendation. One vial contains 10 ml of solution, corresponding to 10,000,000 IU of Vitamin D_3_. Along with warnings about the consequences of an overdose, the producer mentions a decrease in bone mineralization and calcification of some soft tissues. Based on the history of supplementation reported by the breeder, a hypervitaminosis D was suspected to be the most probable cause of the described dystrophic mineralization.

Even though the results of the biochemical serum analyses are consistent with the reported clinical course and seem to confirm the hypothesis of a hypervitaminosis D, it is important to keep in mind that, as stated earlier, post-mortem blood serum values are not totally reliable. Vitamin D intoxication was suspected based on the hypersupplementation history and necropsy and histopathological findings as well as on the hyperphosphatemia, hyperuricemia, hypercreatinemia and hypoproteinemia. The cause of the hyperphosphataemia could be a decreased renal phosphate excretion due to a probable chronic renal insufficiency caused by a potential calcinosis [16]; it is also possible that the increased phosphate levels were associated to the release of phosphorus by the red blood cells following haemolysis [17], given that the blood serum had been sampled from the heart one hour after the animal’s death. The presumed renal failure could also be the cause of the mild hypocalcemia and mild hypoalbuminemia [18]. The detected hyperuricemia (17.3 mmol/l; range 4.5–9.1 mmol/l) could also be closely related to a chronic kidney disease. The hypercreatinemia (288 μmol/l; range 104–168 μmol/l), presumably caused by a reduced renal clearance, and hypoproteinemia (48 g/l; range 56.2–70.4 g/l) seem to prove the hypothesis of renal insufficiency [1, 18]; creatinine clearance has indeed been reported to be lower in individuals who presented renal damage, such as sclerosis of the kidneys [19]. Unfortunately we were unable to perform a histopathological assay on the kidney, which would have been extremely useful in confirming our thesis. A chronic hyperphosphataemia causes initially few complaints and does not upset the general wellbeing of the animal. However, increased phosphate levels lead to extra osseous calcium phosphate deposits in various soft tissues, including arteries, in the joints and connective tissues. The result is a progressive and increasing stenosis of the blood vessels that can lead to cardiac and peripheral circulatory disorders [20, 21, 14, 22]. Proper knowledge of the metabolism and the nutritional needs of these animals are therefore necessary in order to prevent deficiency disorders and nutritional problems. Furthermore, other possible causes of dystrophic mineralization include arterial calcifications in chronic kidney disease [22]; this has been frequently encountered in human medicine, unfortunately we were unable to find, in literature, other causes of dystrophic mineralization in SAC.

Overzealous cholecalciferol (Vitamin D) supplementation is known to lead to Vitamin D toxicity, including tissue mineralization and renal failure, not only in animals but also in humans [20, 21]. A correct process of dosing should always be based on history and on actual body weight of the animal and the patient when administered.

Llamas and alpacas have been subject to much research in the South American and Australian regions because of the great density of such animals in those areas, whereas in Europe most of these individuals have been imported for hobby and other economic factors. Most of the information about SAC is indeed produced in South America, the United States of America and Australia, and is therefore extremely subject to and associated with territorial variations in disease incidence and husbandry practices. Studies based on SAC wellbeing and diseases, as well as other possible treatments should be undertaken in Europe and other countries of the world in order to document the different presentations of disease brought about by the influence of climatic conditions, habitat variations and governmental requirements.

## Abbreviations

HE: Haematoxylin and eosin
IZSVem: Istituto Zooprofilattico Sperimentale delle Venezie
SAC: South American Camelids
